# Dietary vitamin A intake and the risk of ovarian cancer: a meta-analysis

**DOI:** 10.1042/BSR20193979

**Published:** 2020-04-07

**Authors:** Qiaoqiao Wang, Chaying He

**Affiliations:** 1 Zhejiang University City College, No. 51, Huzhou Street, Hangzhou 310015, China; 2 Hangzhou Women's Hospital, No. 369, Kun Peng Road, Shangcheng District, Hangzhou 310008, China

**Keywords:** Dietary intake, Meta-analysis, Ovarian cancer, Vitamin A

## Abstract

**BACKGROUND:** Previous studies have demonstrated some associations between dietary vitamin A intake and ovarian cancer risk with an inconsistent relationship. We therefore performed the present study to further explore the association between them.

**METHODS:** Databases of PubMed, Embase, and Web of Science were retrieved up to September 1, 2019. Summarized relative risk (RR) with corresponding 95% confidence intervals (CI) were calculated. Stata 14.0 software was used for data analysis.

**RESULTS:** Fifteen articles involving 4882 cases and 443,179 participants were included in this meta-analysis. A positive association between dietary vitamin A intake and ovarian cancer risk was found (RR = 0.816, 95%CI = 0.723–0.920, *I*^2^ = 48.4%, *P*_for heterogeneity_ = 0.019). Significant association was also found in case–control studies (RR = 0.769, 95%CI = 0.655–0.902), but not in cohort studies. When we performed the analysis between ovarian cancer risk and geographic locations, we found an inverse association in North American populations (RR = 0.825, 95%CI = 0.720–0.946), instead of other populations.

**CONCLUSIONS:** In summary, findings from the present study suggested that higher dietary intake of vitamin A may contribute to the lower development of ovarian cancer, especially among North Americans.

## Introduction

Ovarian cancer is the most deadly gynecological cancer. The American Cancer Society had estimated that there were 22,240 new cases developing in ovarian cancer and 14,070 ovarian cancer cases deaths in 2018 [1]. Efforts to identify lifestyle factors that may affect the risk of ovarian cancer had been ongoing and indicated that some reproductive factors, such as oral contraceptives, carrying children and tubal ligation, may affect disease risk [2,3]. However, these factors usually cannot be changed. Dietary antioxidants, including vitamin A, have been hypothesized to modify cancer risk [4,5]. A previous study had been published to explore the association about vitamin A consumption and ovarian cancer risk, resulted non-significant association [6]. Many articles about vitamin A intake and ovarian cancer risk had been published, with no consistent conclusion. For this reason, this paper increased the sample size and improved the vitamin A efficiency through a meta-analysis to obtain more authentic and reliable analysis results, which is helpful to clarify whether dietary vitamin A intake has some inverse effects on ovarian cancer development, and finally provides evidence of prevention on ovarian cancer.

## Methods

### Search strategy and inclusion criteria

Three electronic databases (PubMed, Embase and Web of Science) were searched for relevant studies that investigated the association between dietary vitamin A intake and risk of ovarian cancer from inception up to September 1, 2019. The following search terms were used: ‘vitamin A’ OR ‘vitamin^*’^ OR ‘retinol’ combined with ‘ovarian cancer’ OR ‘ovarian tumor’. The bibliographies of the collected studies and relevant reviews were retrospectively assessed to identify additional articles. All the studies enrolled using this strategy was checked independently by two authors.

Studies were included based on the following criteria: (1) patients were diagnosed of ovarian cancer; (2) observational studies; (3) the interested association was about dietary vitamin A or retinol intake and ovarian cancer; (4) available relative risk (RR) and 95% confidence interval (CI) for ovarian cancer.

The following exclusion criteria were used: (1) reviews or meetings or abstracts or letter to the editors; (2) overlap articles or populations; (3) animal studies; (4) no available data of RR and 95%CI; (5) vitamin supplement.

### Data extraction and quality assessment

Two researchers independently reviewed and extracted relevant information from all included studies. These pieces of information included: name of the first author, publishing date, country, ages, study types, vitamin types, sample sizes of the cases and controls, RR and 95%CI for ovarian cancer, adjustment for covariates. The disagreements with these two researchers were resolved by discussion and consensus. The Newcastle–Ottawa Quality Assessment Scale was used to assess the quality of the included studies [7].

### Statistical analysis

Data were summarized using a random-effects model for combined RR with its 95%CI [8]. A *Q* and *I*^2^ test were performed to analysis the heterogeneity of the studies that included in this meta-analysis [9]. Moreover, meta-regression was performed to interpret the between-group heterogeneity [10]. Furthermore, sensitivity analyses were conducted to examine the stability of the results by removing each study one by one. Potential publication biases were examined using Begg’s test and Begg’s funnel plots [11]. Statistical analysis was performed using Stata version 14.0. A two-sided *P* value less than 0.05 was considered statistically significant.

## Results

### Study selection and study characterization

**Figure 1:**
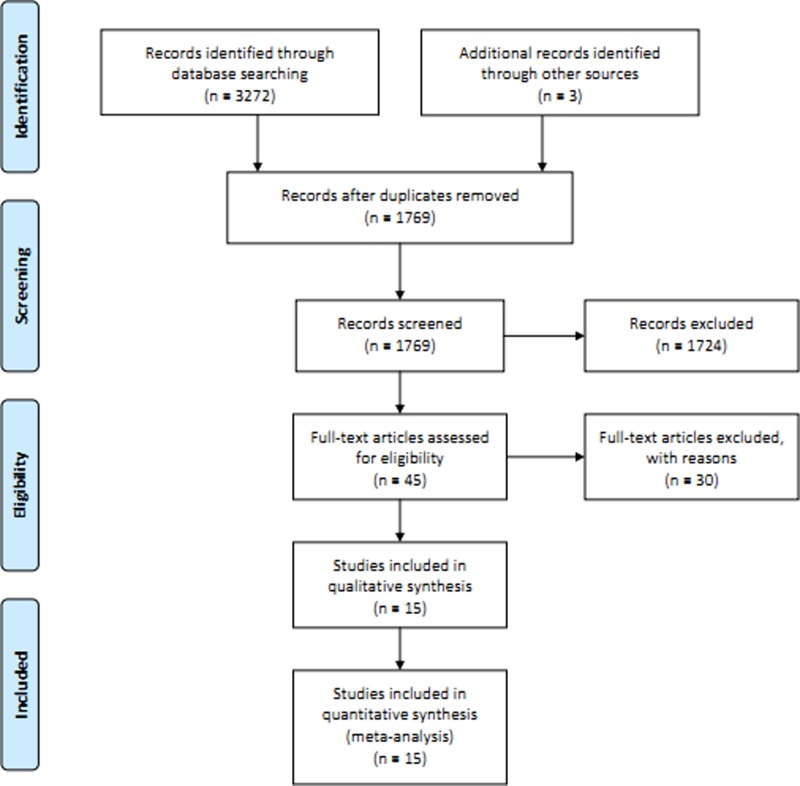
Flow chart of meta-analysis for exclusion/inclusion of studies

After searching the pre-defined three databases, we got 3272 citations. Through removing the duplicated literatures among databases and those obviously did not meet the criteria by reading the title and abstract, 45 full texts of the papers were downloaded for the further screening. Thirty articles were further excluded due to some reasons (duplicate publication (*n* = 1); did not report RR (*n* = 8); reviews (*n* = 8); vitamin A supplement (*n* = 2); animal studies (*n* = 4); serum studies (*n* = 6); assessment of survival rate of ovarian cancer (*n* = 1)). Finally, 15 articles [12–26] were included this meta-analysis (Figure 1). The quality evaluation score (Table 1) of each study ranged from 6 to 8 and the methodological quality was higher. The characteristics of the observational studies are shown in Table 1.

**Table 1:** Characteristics of the included studies about the association of dietary vitamin A intake on ovarian cancer risk

Study, year	Design	Age	Participants, Cases	Country	Vitamin A type	Quality score	RR (95%CI) Highest vs. lowest	Adjustment
Bertone ER, 2001	PBCC	50–79	3,456, 327	United States	Vitamin A	6	0.84(0.57–1.20)	Adjusted for age at interview, state, parity, tubal ligation, and family history of ovarian cancer in a first-degree relative.
Chang ET, 2007	Cohort	<84	97,275, 280	United States	Retinol	7	1.17(0.52–2.66)	Adjusted for race, total energy intake, parity, oral contraceptive use, strenuous exercise, wine consumption, and menopausal status/hormone therapy use; stratified by age at baseline.
Cramer DW, 2001	PBCC	>50	1,065, 549	United States	Vitamin A	7	0.60(0.39–0.94)	Adjusted for total caloric intake, age, site, parity, body mass index, oral contraceptive use, family history of breast, ovarian or prostate cancer in a first-degree relative, tubal ligation, education and marital status.
Fairfield KM, 2001	Cohort	30–55	80,326, 301	United States	Vitamin A	7	0.86(0.60–1.23)	Adjusted for age, body mass index (kg/m^2^), duration of oral contraception use, smoking history, parity, history of tubal ligation, and caffeine intake.
Kushi LH, 1999	Cohort	55–69	29,083, 139	United States	Vitamin A	6	1.11(0.65–1.88)	Adjusted for age, total energy intake, number of live births, age at menopause, family history of ovarian cancer in a first-degree relative, hysterectomy/ unilateral oophorectomy status, waist-to-hip ratio, level of physical activity, cigarette smoking (number of pack-years), and educational level.
La Vecchia C, 1987	HBCC	22–74	1,840, 455	Italy	Vitamin A	7	0.94(0.72–1.22)	Adjusted for age (in cardinal form), interviewer, marital status, social class, education, parity, age at first birth, age at menarche, menopausal status, age at menopause, body mass index, and oral contraceptive and other female hormone use.
McCann SE, 2001	HBCC	20–87	1,921, 496	United States	Vitamin A	8	0.66(0.45–0.98)	Adjusted for age, education, region of residence, regularity of menstruation, family history of ovarian cancer, parity, age at menarche, oral contraceptive use, and total energy intake.
Risch HA, 1994	PBCC	35–79	1,014, 450	Canada	Retinol	7	1.00(0.92–1.09)	Adjusted for age at diagnosis/ interview and the continuous variables age, total daily calorie intake, number of full-term pregnancies, and total duration of oral contraceptive use. Each line in this table represents two individual models.
Salazar-Martinez E, 2002	HBCC	20–79	713, 84	Mexico	Retinol	8	0.52(0.28–0.95)	Adjusted for age, total energy intake, number of live births, recent changes in weight, physical activity and diabetes.
Silvera SA, 2006	Cohort	40–59	89,835, 264	Canada	Vitamin A	8	0.77(0.52–1.14)	Adjusted for age, menopausal status, use of oral contraceptives, body mass index, education, participation in vigorous physical activity, energy intake at baseline, study center, and randomization group.
Slattery ML, 1989	PBCC	20–79	577, 85	United States	Vitamin A	6	0.7(0.4–1.3)	Adjusted for age, body mass index of weight/height^2^, and number of pregnancies. All dietary variables are in separate logistic models.
Thomson CA, 2008	Cohort	50–79	133,614, 451	United States	Vitamin A	8	0.91(0.62–1.32)	Adjusted for age, log calories, No. breast/ovary cancer relatives, dietary modification randomization arm, hysterectomy status, minority race, pack-years smoking, physical activity, nonsteroidal anti-inflammatory drug use, parity, infertility, duration of oral contraceptive use, lifetime ovulatory cycles, partial oophorectomy, age at menopause, and HT usage at entry.
Tung KH, 2005	PBCC	45–75	1,165, 558	United States	Vitamin A	7	0.72(0.49–1.07)	Adjusted for age, ethnicity, study site, education, oral contraceptive pill use, pregnancy status, tubal ligation, and energy intake by polytomous logistic regression (histologic type) or unconditional logistic regression (all other variables)
Tzonou A, 1993	HBCC	18–75	389, 189	Greece	Vitamin A	7	0.87(0.73–1.03)	Adjusted for age, years of schooling, parity, age at first birth, menopausal status as well as for energy intake.
Zhang M, 2004	HBCC	18–75	906, 254	China	Vitamin A	8	0.41(0.24–0.69)	Adjusted for terms for age, locality, education, family income, BMI, total energy intake, tobacco smoking, alcohol consumption, ovarian cancer in first degree relatives, parity, menopausal status, and oral contraceptive use.

Abbreviations: CI, confidence interval; HBCC, hospital-based case–control study; PBCC, population-based case–control study; RR, relative risk.

### Dietary vitamin A intake and the risk of ovarian cancer

**Figure 2:**
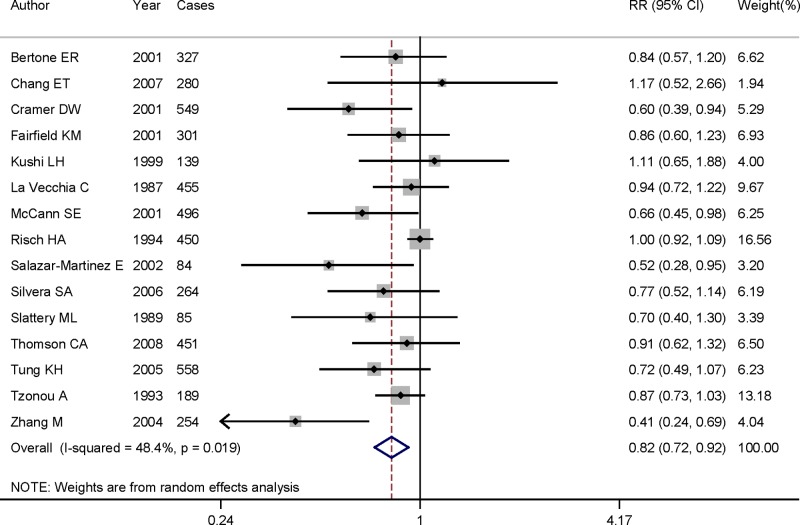
The forest plot of the relationship between dietary vitamin A intake and ovarian cancer risk

Pooled RR suggested that highest category of dietary vitamin A intake could significantly reduce the risk of ovarian cancer (RR = 0.816, 95%CI = 0.723–0.920, *I*^2^ = 48.4%, *P*_for heterogeneity_ = 0.019) (Figure 2), when compared with the lowest category. In the included studies, five studies were cohort design and the remaining 10 studies were case–control design. Significant association was also found in case–control studies (RR = 0.769, 95%CI = 0.655–0.902), but not in cohort studies (RR = 0.895, 95%CI = 0.736–1.088). When we performed the subgroup analysis between ovarian cancer risk and geographic locations, we found an inverse association in North American populations (RR = 0.825, 95%CI = 0.720–0.946), instead of European populations (RR = 0.890, 95%CI = 0.771–1.028). We did not assess the association between dietary vitamin A intake and the risk of ovarian cancer while only one study was from Asian population. Table 2 shows the results for both whole and subgroup analyses.

**Table 2:** Summary RR and 95%CI of the association between dietary vitamin A intake and ovarian cancer risk

Subgroups	Number of studies	Number of cases	RR(95% CI)	*P* for trend	Heterogeneity test	
					*I*^2^ (%)	*P*
Overall	15	4882	0.816(0.723–0.920)	0.001	48.4	0.019
Study design						
Cohort	5	1435	0.895(0.736–1.088)	0.264	0.0	0.797
Case–control	10	3447	0.769(0.655–0.902)	0.001	64.6	0.003
PBCC	5	1969	0.812(0.651–0.994)	0.037	55.3	0.062
HBCC	5	1478	0.712(0.551–0.922)	0.010	64.8	0.023
Geographic locations						
North America	12	3984	0.825(0.720-0.946)	0.006	38.4	0.088
Europe	2	644	0.890(0.771–1.028)	0.114	0.0	0.630
Asia	1	254	–	–	–	–

Abbreviations: CI, confidence interval; HBCC, hospital-based case–control studies; PBCC, population-based case–control studies; RR, relative risk.

### Publication bias and sensitivity analysis

**Figure 3:**
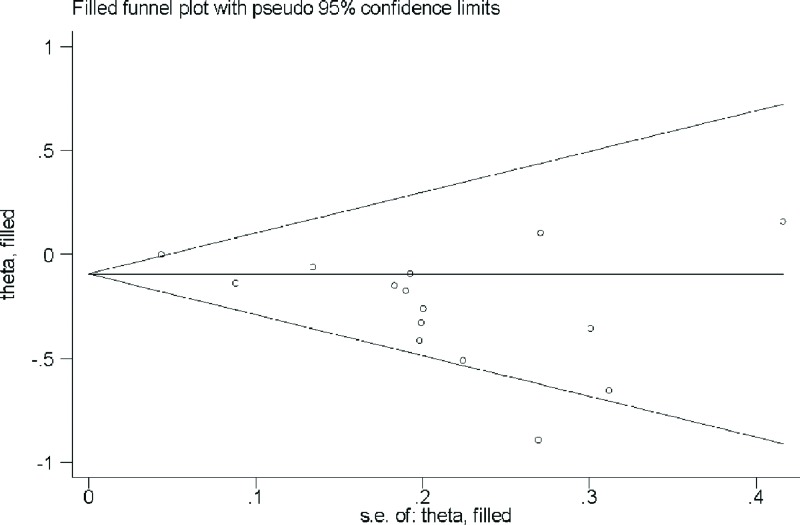
Funnel plot for the analysis of publication bias between dietary vitamin A intake and ovarian cancer risk

**Figure 4:**
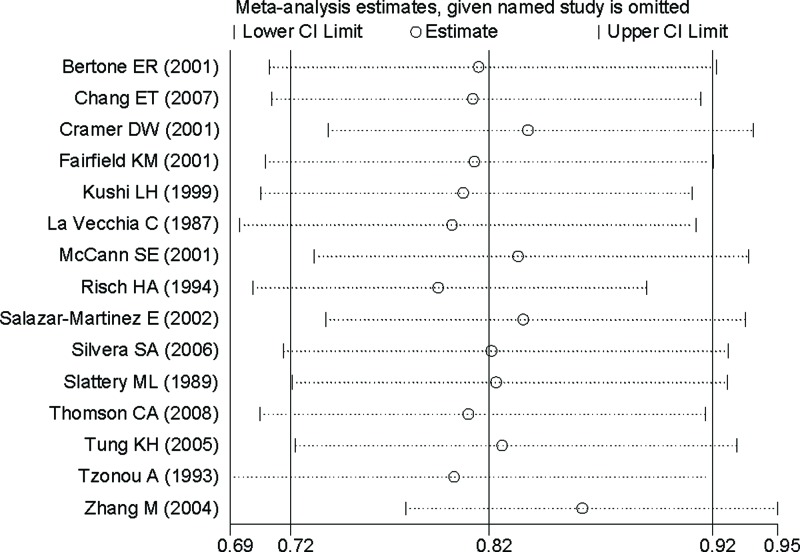
Sensitivity analyses between dietary vitamin A intake and ovarian cancer risk

Based on Begg’s test (*P* = 0.318) and funnel plot (Figure 3), there existed no publication bias. Sensitivity analysis showed that no single study had a potential impact on the pooled RR (Figure 4).

## Discussion

Our findings based on 15 studies obtained that highest category of dietary vitamin A intake could significantly reduce the risk of ovarian cancer, when compared with the lowest category. We also found a significant association in case–control studies. An inverse association was found in North American populations, instead of other populations.

Our meta-analysis was different from the previous meta-analysis by Koushik et al. [6]. First, although the authors included 10 cohort studies, they said only 4 studies had previously published on vitamin consumption and ovarian cancer risk, in which only 3 studies were about vitamin A and ovarian cancer risk. However, we included 15 articles to explore the association between vitamin A intake and the risk of ovarian cancer. Second, the authors only included cohort studies, which may omit many observational studies. However, we included both cohort studies and case–control studies in our meta-analysis. Third, they concluded that consumption of vitamin A during adulthood does not play a major role in ovarian cancer risk. However, findings from our meta-analysis suggested that dietary vitamin A intake could reduce the risk of ovarian cancer. Interestingly, we obtained a consistent result in the cohort studies although we included 5 cohort studies, which is two more than Koushik et al.

Previous meta-analyses had been published to assess the intake of vitamin A and cancer risk. Huang et al. and Zhang et al. had explored the association between vitamin A intake and pancreatic cancer [27,28], they obtained a consistent result, which might inversely correlate with pancreatic cancer while with vitamin A intake. Yu et al. concluded that higher category of dietary vitamin A intake could reduce the risk of lung cancer [29]. Lv et al. also concluded an inverse association between vitamin A intake and glioma risk [30]. Moreover, Kong et al. found that dietary vitamin A had a significant reduction in the risk of gastric cancer [31]. Our results are all consistent with the previous meta-analyses.

We found significant between-study heterogeneity in the whole pooled results of dietary vitamin A intake and ovarian cancer risk. Between-study heterogeneity in the meta-analysis is common, and it is an essential component to explore the heterogeneity existed in the between-study. Meta-regression was used to explore the causes of heterogeneity for covariates of publication year, vitamins type, study design, geographic locations and number of cases. Results from meta-regression suggested that geographic locations (*P* = 0.021) was significantly associated with this high heterogeneity. When we performed the subgroup analysis by geographic locations, the *I*^2^ was reduced to 38.4% in North American populations and 0.0% in European populations. The result in subgroup of North American populations was consistent with the whole pooled result, while 12 of the 15 included studies were from North America.

However, several limitations should be attention. First, only English language articles were included, which may omit other languages studies. Furthermore, we only searched the articles which had been published in the journal, and did not be able including the meeting articles and some unpublished articles. However, we did not detect any publication bias, suggested our results were stable. Second, 10 of the 15 studies were case–control studies and only 5 were cohort studies. The selection bias, recall bias and some other confounding factors cannot be excluded; for example, some subjects may change their dietary vitamin A intake after the baseline assessment. However, case–control design was a very important epidemiological approach in the observational study. Therefore, it is requirement for evidence from prospective cohort studies. Third, 12 of the 15 studies were from North America, and the result was consistent with the whole pooled result. However, only 2 studies were from Europe and 1 study from Asia. We did not obtain an inverse association between dietary vitamin A intake and the risk of ovarian cancer in Europeans and Asians. Therefore, the result in the present study was more suitable for North America, but not for any populations else. Thus, more studies conducted in some other populations, instead of North Americans, are warranted to further explore the relationship between dietary vitamin A intake and the risk of ovarian cancer.

## Conclusions

In summary, our results concluded that dietary intake of vitamin A may contribute to the lower development of ovarian cancer, especially among North Americans. As some limitations existed in our analysis, large scale studies with detailed amount of dietary vitamin A intake are needed to verify our results.

## Abbreviations

CI: confidence interval
HBCC: hospital-based case–control study
PBCC: population-based case–control study
RR: relative risk
